# 1,4-Bis(fluoro­meth­yl)benzene

**DOI:** 10.1107/S1600536809003730

**Published:** 2009-02-04

**Authors:** Hoong-Kun Fun, Reza Kia, P. S. Patil, S. M. Dharmaprakash

**Affiliations:** a X-ray Crystallography Unit, School of Physics, Universiti Sains Malaysia, 11800 USM, Penang, Malaysia; b Department of Physics, K. L. E. Society’s K. L. E. Institute of Technology, Gokul, Hubli 580 030, India; c Department of Studies in Physics, Mangalore University, Mangalagangotri, Mangalore 574 199, India

## Abstract

The title compound, C_8_H_8_F_2_, lies across a crystallographic inversion centre. The structure features short C⋯F [2.8515 (18) Å] and F⋯F [2.490 (4) Å] contacts, which are significantly shorter than the sum of the van der Waals radii of these atoms. The F atom and methyl­ene H atoms are disordered over two positions with a site-occupancy ratio of 0.633 (3):0.367 (3). In the crystal structure, inter­molecular C—H⋯F inter­actions link neighboring mol­ecules into infinite chains along the *b* axis. In addition, C—H⋯π inter­actions link these mol­ecules along [10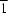], forming a two-dimensional network parallel to (101).

## Related literature

For the structures of compounds with non-linear properties, see, for example: Chantrapromma *et al.* (2006 ▶); Fun *et al.* (2008 ▶); Patil *et al.* (2007 ▶).
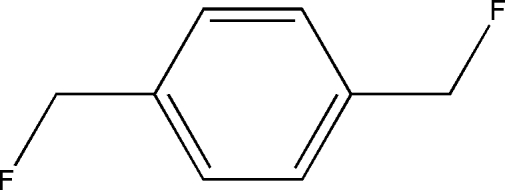


## Experimental

### 

#### Crystal data

C_8_H_8_F_2_
*M* *_r_* = 143.15
Monoclinic, 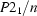

*a* = 6.1886 (2) Å
*b* = 5.0152 (2) Å
*c* = 10.4750 (4) Å
β = 95.107 (2)°
*V* = 323.82 (2) Å^3^
*Z* = 2
Mo *K*α radiation
μ = 0.12 mm^−1^
*T* = 100.0 (1) K
0.55 × 0.24 × 0.14 mm

#### Data collection

Bruker APEXII CCD area-detector diffractometer
Absorption correction: multi-scan (**SADABS**; Bruker, 2005 ▶) *T*_min_ = 0.935, *T*_max_ = 0.982
11592 measured reflections
1591 independent reflections
1343 reflections with *I* > 2σ(*I*)
*R*_int_ = 0.029

#### Refinement

*R*[*F* ^2^ > 2σ(*F* ^2^)] = 0.074
*wR*(*F* ^2^) = 0.251
*S* = 1.18
1591 reflections
64 parameters
H atoms treated by a mixture of independent and constrained refinement
Δρ_max_ = 0.67 e Å^−3^
Δρ_min_ = −0.59 e Å^−3^

### 

Data collection: *APEX2* (Bruker, 2005 ▶); cell refinement: *SAINT* (Bruker, 2005 ▶); data reduction: *SAINT*; program(s) used to solve structure: *SHELXTL* (Sheldrick, 2008 ▶); program(s) used to refine structure: *SHELXTL*; molecular graphics: *SHELXTL*; software used to prepare material for publication: *SHELXTL* and *PLATON* (Spek, 2003 ▶).

## Supplementary Material

Crystal structure: contains datablocks global, I. DOI: 10.1107/S1600536809003730/bq2122sup1.cif

Structure factors: contains datablocks I. DOI: 10.1107/S1600536809003730/bq2122Isup2.hkl

Additional supplementary materials:  crystallographic information; 3D view; checkCIF report

## Figures and Tables

**Table 1:** Hydrogen-bond geometry (Å, °)

*D*—H⋯*A*	*D*—H	H⋯*A*	*D*⋯*A*	*D*—H⋯*A*
C4—H4*D*⋯F1*A*^i^	0.96	2.04	2.8515 (18)	141
C4—H4*B*⋯*Cg*1^ii^	0.97	2.84	3.5148 (12)	128
C4—H4*C*⋯*Cg*1^ii^	0.96	2.64	3.5148 (12)	144

Symmetry codes: (i) 
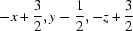
; (ii) 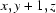. *Cg*1 is the centroid of the C1–C3/C1*A*–C3*A* benzene ring.

**Fig. 1.**
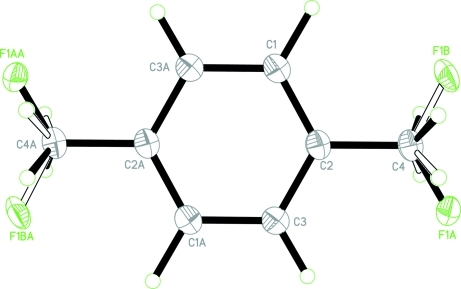
The molecular structure of the title compound, showing 50% probability displacement ellipsoids and the atomic numbering. Open bonds indicate the minor disordered component.

**Fig. 2.**
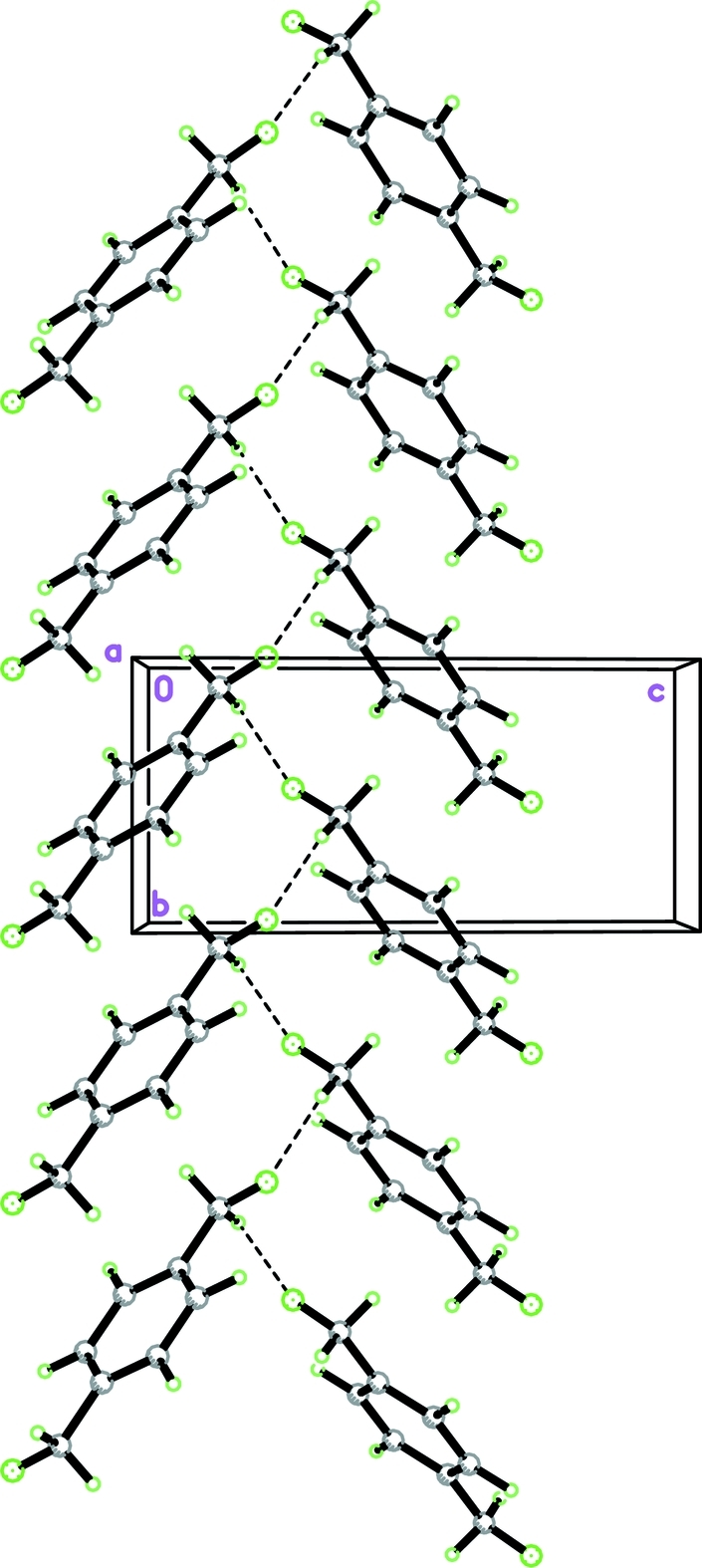
The crystal packing of the major component of (I), viewed down the a-axis, showing a one-dimensional infinite chain of molecules along the b-axis. Intramolecular and intermolecular interactions are drawn as dashed lines.

## supplementary crystallographic information

### Comment

As part of an ongoing investigation into compounds with non-linear optical properties (Chantrapromma *et al.*, 2006; Fun *et al.*, 2008; Patil *et al.*, 2007), the crystal structure of the title compound is presented here.

The title compound, (I), lies across a crystallographic inversion centre (Fig. 1). The interesting features of the crystal structure are the short C4A···F1A^i^ [2.8515 (18)Å; (i) 3/2-*x*, 1/2+*y*, 3/2-*z*] and F1B···F1B^ii^ [2.490 (4)Å; (ii) 1-*x*, 1-*y*, 1-*z*] contacts which are significantly shorter than the sum of the van der Waals radii of these atoms. The fluorine atom and methylene hydrogens are disordered over two positions with a site-occupancy ratio of 0.633 (3):0.367 (3). In the crystal structure, intermolecular C—H···F interactions link neighboring molecules into one-dimensional infinite chains along the *b* axis (Table 1 and Fig. 2). In addition, C—H···π interactions [C4—H4B···*Cg*1^iii^; (iii) *x*, 1+*y*, *z* and C4—H4C···*Cg*1^iii^; *Cg*1 is the centroid of the C1–C3/C1A–C3A benzene ring] link these molecules along the [101] direction, thus forming a two-dimensional network which is parallel to the (101) plane.

### Experimental

Commercially available 1,4-bis(difluoromethyl) benzene was further purified by repeated recrystallization from acetone. Single crystals suitable for X-ray analysis were grown by slow evaporation of an acetone solution at room temperature.

### Refinement

The hydrogen atoms bound to C1 and C3 were located from the difference Fourier map and refined freely. Hydrogen atoms of the methylene groups were positioned geometrically and constrained to refine with a riding model approximation with C—H = 0.96–0.97 Å and *U*_iso_(H) = 1.2 *U*_eq_(C).

### Crystal data

**Table:**

C_8_H_8_F_2_	*F*(000) = 148
*M**_r_* = 143.15	*D*_x_ = 1.458 Mg m^−^^3^
Monoclinic, *P*2_1_/*n*	Mo *K*α radiation, λ = 0.71073 Å
Hall symbol: -P 2yn	Cell parameters from 3653 reflections
*a* = 6.1886 (2) Å	θ = 2.5–34.7°
*b* = 5.0152 (2) Å	µ = 0.12 mm^−^^1^
*c* = 10.4750 (4) Å	*T* = 100 K
β = 95.107 (2)°	Needle, colourless
*V* = 323.82 (2) Å^3^	0.55 × 0.24 × 0.14 mm
*Z* = 2	

### Data collection

**Table:**

Bruker APEXII CCD area-detector diffractometer	1591 independent reflections
Radiation source: fine-focus sealed tube	1343 reflections with *I* > 2σ(*I*)
graphite	*R*_int_ = 0.029
φ and ω scans	θ_max_ = 36.6°, θ_min_ = 3.7°
Absorption correction: multi-scan (*SADABS*; Bruker, 2005)	*h* = −10→10
*T*_min_ = 0.935, *T*_max_ = 0.982	*k* = −7→8
11592 measured reflections	*l* = −17→17

### Refinement

**Table:**

Refinement on *F*^2^	Primary atom site location: structure-invariant direct methods
Least-squares matrix: full	Secondary atom site location: difference Fourier map
*R*[*F*^2^ > 2σ(*F*^2^)] = 0.074	Hydrogen site location: inferred from neighbouring sites
*wR*(*F*^2^) = 0.251	H atoms treated by a mixture of independent and constrained refinement
*S* = 1.18	*w* = 1/[σ^2^(*F*_o_^2^) + (0.1441*P*)^2^ + 0.1329*P*] where *P* = (*F*_o_^2^ + 2*F*_c_^2^)/3
1591 reflections	(Δ/σ)_max_ < 0.001
64 parameters	Δρ_max_ = 0.67 e Å^−^^3^
0 restraints	Δρ_min_ = −0.59 e Å^−^^3^

### Special details

**Table:**

Experimental. The low-temperature data was collected with the Oxford Cyrosystem Cobra low-temperature attachment.
Geometry. All e.s.d.'s (except the e.s.d. in the dihedral angle between two l.s. planes) are estimated using the full covariance matrix. The cell e.s.d.'s are taken into account individually in the estimation of e.s.d.'s in distances, angles and torsion angles; correlations between e.s.d.'s in cell parameters are only used when they are defined by crystal symmetry. An approximate (isotropic) treatment of cell e.s.d.'s is used for estimating e.s.d.'s involving l.s. planes.
Refinement. Refinement of *F*^2^ against ALL reflections. The weighted *R*-factor *wR* and goodness of fit *S* are based on *F*^2^, conventional *R*-factors *R* are based on *F*, with *F* set to zero for negative *F*^2^. The threshold expression of *F*^2^ > σ(*F*^2^) is used only for calculating *R*-factors(gt) *etc*. and is not relevant to the choice of reflections for refinement. *R*-factors based on *F*^2^ are statistically about twice as large as those based on *F*, and *R*- factors based on ALL data will be even larger.

### Fractional atomic coordinates and isotropic or equivalent isotropic displacement parameters (Å^2^)

**Table:**

	*x*	*y*	*z*	*U*_iso_*/*U*_eq_	Occ. (<1)
F1A	0.9163 (2)	0.5267 (3)	0.72717 (12)	0.0219 (3)	0.633 (3)
F1B	0.6221 (4)	0.4849 (5)	0.6008 (3)	0.0260 (6)	0.367 (3)
C4	0.8122 (2)	0.4155 (2)	0.63866 (12)	0.0204 (3)	
H4C	0.7640	0.5505	0.5776	0.025*	0.633 (3)
H4D	0.6847	0.3437	0.6718	0.025*	0.633 (3)
H4A	0.8129	0.3600	0.7274	0.025*	0.367 (3)
H4B	0.9052	0.5709	0.6369	0.025*	0.367 (3)
C1	0.7876 (2)	0.0844 (3)	0.46363 (13)	0.0222 (3)	
C2	0.9095 (2)	0.1998 (2)	0.56704 (11)	0.0194 (3)	
C3	1.1209 (2)	0.1184 (3)	0.60446 (12)	0.0217 (3)	
H1	0.620 (4)	0.159 (6)	0.431 (2)	0.037 (6)*	
H3	1.207 (4)	0.205 (5)	0.682 (2)	0.026 (5)*	

### Atomic displacement parameters (Å^2^)

**Table:**

	*U*^11^	*U*^22^	*U*^33^	*U*^12^	*U*^13^	*U*^23^
F1A	0.0227 (6)	0.0216 (6)	0.0215 (6)	0.0012 (4)	0.0030 (4)	−0.0057 (4)
F1B	0.0216 (10)	0.0244 (11)	0.0324 (12)	0.0115 (8)	0.0045 (8)	−0.0018 (8)
C4	0.0224 (5)	0.0170 (5)	0.0227 (5)	0.0024 (4)	0.0064 (4)	0.0013 (4)
C1	0.0204 (5)	0.0220 (5)	0.0243 (5)	0.0030 (4)	0.0023 (4)	0.0001 (4)
C2	0.0208 (5)	0.0176 (5)	0.0204 (5)	0.0020 (3)	0.0048 (4)	0.0013 (4)
C3	0.0207 (5)	0.0217 (6)	0.0226 (5)	0.0016 (4)	0.0007 (4)	−0.0006 (4)

### Geometric parameters (Å, °)

**Table:**

F1A—C4	1.2162 (18)	C4—H4A	0.9699
F1A—H4A	1.0529	C4—H4B	0.9700
F1A—H4B	0.9681	C1—C2	1.3900 (18)
F1B—C4	1.257 (3)	C1—C3^i^	1.3916 (19)
F1B—H4C	0.9881	C1—H1	1.13 (3)
F1B—H4D	1.0739	C2—C3	1.3927 (18)
C4—C2	1.4754 (17)	C3—C1^i^	1.3916 (19)
C4—H4C	0.9600	C3—H3	1.02 (2)
C4—H4D	0.9600		
			
C4—F1A—H4A	50.0	C2—C4—H4A	108.0
C4—F1A—H4B	51.2	H4C—C4—H4A	144.6
H4A—F1A—H4B	101.2	H4D—C4—H4A	58.7
C4—F1B—H4C	48.9	F1A—C4—H4B	51.1
C4—F1B—H4D	47.9	F1B—C4—H4B	108.2
H4C—F1B—H4D	96.7	C2—C4—H4B	108.0
F1A—C4—F1B	122.09 (16)	H4C—C4—H4B	64.6
F1A—C4—C2	120.74 (12)	H4D—C4—H4B	144.8
F1B—C4—C2	117.11 (16)	H4A—C4—H4B	107.3
F1A—C4—H4C	107.2	C2—C1—C3^i^	119.09 (12)
F1B—C4—H4C	50.8	C2—C1—H1	121.3 (14)
C2—C4—H4C	107.2	C3^i^—C1—H1	119.6 (14)
F1A—C4—H4D	107.0	C1—C2—C3	121.91 (12)
F1B—C4—H4D	56.0	C1—C2—C4	118.92 (11)
C2—C4—H4D	107.1	C3—C2—C4	119.17 (12)
H4C—C4—H4D	106.8	C1^i^—C3—C2	119.00 (12)
F1A—C4—H4A	56.2	C1^i^—C3—H3	120.6 (14)
F1B—C4—H4A	107.9	C2—C3—H3	120.4 (14)
			
C3^i^—C1—C2—C3	0.1 (2)	F1A—C4—C2—C3	1.96 (19)
C3^i^—C1—C2—C4	179.75 (11)	F1B—C4—C2—C3	179.18 (17)
F1A—C4—C2—C1	−177.73 (13)	C1—C2—C3—C1^i^	−0.1 (2)
F1B—C4—C2—C1	−0.5 (2)	C4—C2—C3—C1^i^	−179.75 (11)

Symmetry codes: (i) −*x*+2, −*y*, −*z*+1.

### Hydrogen-bond geometry (Å, °)

**Table:**

*D*—H···*A*	*D*—H	H···*A*	*D*···*A*	*D*—H···*A*
C4—H4D···F1A^ii^	0.96	2.04	2.8515 (18)	141
C4—H4B···Cg1^iii^	0.97	2.84	3.5148 (12)	128
C4—H4C···Cg1^iii^	0.96	2.64	3.5148 (12)	144

Symmetry codes: (ii) −*x*+3/2, *y*−1/2, −*z*+3/2; (iii) *x*, *y*+1, *z*.
